# CTLA-4 antibody ipilimumab negatively affects CD4^+^ T-cell responses in vitro

**DOI:** 10.1007/s00262-019-02369-x

**Published:** 2019-07-22

**Authors:** Sandra Rosskopf, Judith Leitner, Gerhard J. Zlabinger, Peter Steinberger

**Affiliations:** 1 Division of Immune Receptors and T Cell Activation, Institute of Immunology, Center for Pathophysiology, Infectiology and Immunology, Medical University of Vienna, Lazarettgasse 19, Vienna, Austria; 2 Division of Clinical and Experimental Immunology, Institute of Immunology, Center for Pathophysiology, Infectiology and Immunology, Medical University of Vienna, Vienna, Austria

**Keywords:** Immune checkpoints, Coinhibitory pathways, CTLA-4, Ipilimumab, Tetanus toxoid

## Abstract

Immune checkpoint inhibitors targeting coinhibitory pathways in T cells possess efficacy in combating cancer. In addition to PD-1/PD-L1 and CTLA-4 antibodies which are already established in tumor immunotherapy, immune checkpoints such as LAG-3 or BTLA are emerging, which may have the potential to enhance T-cell responses alone or in combination with PD-1 blockers. CD4^+^ T cells play a central role in the immune system and contribute to productive immune responses in multiple ways. The effects of immune checkpoint inhibitors on this cell subset may thus critically influence therapeutic outcomes. Here, we have used in vitro responses to tetanus toxoid (TT) as a model system to study the effects of immune checkpoint inhibitors on CD4^+^ T-cell responses. CFSE-labeled PBMCs of 65 donors were stimulated with TT in the presence of blocking antibodies to PD-L1, CTLA-4, LAG-3, or BTLA for 7 days. We found that the PD-L1 antibody greatly enhanced cytokine production and antigen-specific CD4^+^ T-cell proliferation, whereas blocking antibodies to BTLA or LAG-3 did not augment responses to TT. Surprisingly, the presence of the therapeutic CTLA-4 antibody ipilimumab resulted in a significant reduction of CD4^+^ T-cell proliferation and cytokine production. Stimulation experiments with an IgG4 variant of ipilimumab indicated that the inhibitory effect of ipilimumab was dependent on its IgG1 isotype. Our results indicate that the therapeutic CTLA-4 antibody ipilimumab can impair CD4^+^ effector T-cell responses and that this activity is mediated by its Fc part and CD16-expressing cells.

**Electronic supplementary material:**

The online version of this article (10.1007/s00262-019-02369-x) contains supplementary material, which is available to authorized users.

## Introduction

T-cell-expressed coinhibitory receptors act as essential immune checkpoints to prevent aberrant activation, thereby maintaining peripheral tolerance. However, they also impair productive immunity in response to pathogens and tumor cells. Blockade of the PD-1/PD-L axis and CTLA-4 has been shown to induce durable responses in patients suffering from different tumors including melanoma and lung cancer [1–3]. T cells harbor additional inhibitory receptors that are considered as potential targets in cancer therapy. Studies in murine tumor models have demonstrated that blockade of lymphocyte-activation gene 3 (LAG-3) alone or in combination with PD-1 antibodies limits tumor growth and promotes clearance of malignant cells [4–7]. Several LAG-3 antibodies and a bispecific agent that concomitantly binds to LAG-3 and PD-1 are currently in clinical development [8]. Another promising target is B- and T-lymphocyte attenuator (BTLA), which is broadly expressed on human T cells and transduces strong inhibitory signals upon engagement by its ligand herpesvirus entry mediator (HVEM). Several studies including the work by our group have revealed that blocking antibodies to this molecule can enhance human T-cell responses when used alone or in combination with PD-1 antibodies [9–11]. In addition, this receptor is robustly expressed in the tumor microenvironment and can function to inhibit tumor-specific human T cells [12].

Studies on immune checkpoints have focused on CD8^+^ T cells since they are the major effectors participating in anti-tumor immunity. However, these molecules are also expressed on CD4^+^ effector T cells, which provide help to other immune cells, augmenting immunity at several levels. Importantly, CD4^+^ T cells can also promote cytotoxicity, e.g., by killing target cells in a MHC class II-dependent or -independent fashion, or by licensing DC to effectively activate cytotoxic CD8^+^ T cells [13, 14].

Here, tetanus toxoid (TT) stimulation was used as a robust in vitro model for analyzing human CD4^+^ T-cell responses to address the stimulatory capacity of immune checkpoint inhibitors targeting PD-L1, CTLA-4, LAG-3, and BTLA. We found that only the blockade of PD-L1 effectively enhanced the response to TT, while LAG-3 and BTLA antibodies had no effect. Surprisingly, addition of the therapeutic CTLA-4 antibody ipilimumab significantly reduced cytokine production and CD4^+^ T-cell proliferation. Ipilimumab is an IgG1 antibody and can, therefore, efficiently interact with Fc receptors. Several recent studies have indicated that ipilimumab might function at least in part by depleting intra-tumoral CTLA-4^high^ Tregs via Fc receptor-dependent mechanisms [15–18]. We observed reduced numbers of proliferated CD4^+^ T cells in the presence of IgG1-ipilimumab but not with an IgG4 variant, indicating that impairment of CD4^+^ T-cell responses by ipilimumab is mediated by its Fc part. Moreover, we demonstrate that depleting CD16^+^ cells abrogated the inhibitory effects of ipilimumab.

## Materials and methods

### Donor collection and PBMC isolation

Thirty-five female and 30 male individuals with a mean age of 35.5 years (range from 19.6 to 61.2) participated in the study. Heparinized whole blood samples were collected to isolate peripheral blood mononuclear cells (PBMCs) by standard gradient density centrifugation with Lymphoprep^®^ solution (Technoclone, Austria).

### Proliferation assay

Carboxyfluorescein succinimidyl ester (CFSE) labeling was performed as described previously [11]. CFSE-labeled PBMCs (1 × 10^5^/well) were stimulated with TT (10 Lf/mL; Statens Serum Institut, Copenhagen, Denmark) in AIM V™ media (Thermo Fisher Scientific, Waltham, MA, USA) supplemented with 1.5% human serum. Blocking antibodies to immune checkpoints were used at a final concentration of 8 µg/mL. After 6–7 days, percentage of CFSE^low^ CD4^+^ T lymphocytes was analyzed by flow cytometry. A single data point represents the triplicate mean of a donor. Responses with a stimulation index of 1.5 (at least 1.5-fold increase in the percentage of CFSE^low^ CD4^+^ T cells in TT-stimulated cultures with respect to the CFSE^low^ CD4^+^ T cells in control cultures) were considered reactive and used for further analysis.

### Cell culture, antibodies, and flow cytometry

For flow cytometry analysis, the monoclonal antibodies CD4-PE (OKT4), BTLA-PE (MIH26), PD-1-PE (EH12.2H7), and isotype control (MOPC-21) were obtained from Biolegend (San Diego, CA, USA). CTLA-4-PE (14D3) and LAG-3-PE (3DS223H) monoclonal antibodies were obtained from eBioscience (San Diego, CA, USA). Staining was performed in FACS buffer (1% BSA and 0.1% NaN_3_ in PBS) for 30 min and 10 mg/mL Beriglobin (CSL Behring, King of Prussia, PA, USA) was added to prevent non-specific binding to Fc receptors while staining of the immune checkpoints. 7-Aminoactinomycin (7-AAD; Biolegend) was used to exclude dead cells from analysis. To determine CTLA-4 expression, cells were permeabilized using the Cytofix/Cytoperm™ kit (BD Biosciences, Franklin Lakes, NJ, USA). For depletion of CD16-expressing cells, PBMCs were labeled with CD16-APC mAb (3G8, Biolegend). The CD16-negative population was isolated using an SH800S cell sorter (Sony Biotechnology, Japan). The purity of the isolated population was examined by flow cytometry.

The following monoclonal blocking antibodies (final concentration 8 μg/mL) were used: functional grade PD-L1 (29E.2A3; LEAF™) and PD-1 (EH12.2H7 LEAF™) from Biolegend, CTLA-4 (IgG1, Ipilimumab, Yervoy^®^), CTLA-4 (Ipilimumab-IgG4, S228P, InvivoGen, San Diego, CA, USA), and a mouse IgG1 κ isotype control antibody (MOPC-21; LEAF™, Biolegend). BTLA and LAG-3 blocking antibodies were described previously [10, 11]. FACSCalibur™ and LSRFortessa™ flow cytometers (BD Bioscience) were used for sample measurement, and the FlowJo software (version 10.4.1., Tree Star, Ashland, OR, USA) was used for flow cytometry data analysis.

### LEGENDplex™ and Luminex-based cytokine analysis

At days 6–7 of the PBMC proliferation assays, culture supernatants were collected from the triplicates, pooled and stored at − 20 °C. Next, IL-5, IL-10, IL-13, IL-17F, IFN-γ, and TNF-α concentrations were measured using the LEGENDplex™ human T_H_ cytokine panel (13-plex, Biolegend). Cytokine data shown in Fig. 6 were obtained by measuring the IFN-γ and TNF-α concentrations in culture supernatants using the Luminex System 100 (Luminex, Texas, USA) as described [19].

### Statistics

Normalization of proliferation and cytokine data was performed using methods described previously [20]. GraphPad Prism 7 (GraphPad Software Inc., La Jolla, CA, USA) was used to perform statistical analyses. Red markings in the figures represent median values of proliferation data, cytokine concentrations, and expression levels. Wilcoxon–Mann–Whitney tests and non-parametric repeated measurement ANOVA (Friedman test) were performed to analyze receptor expression, proliferation, and cytokine data for two or more groups in relation to mock control conditions, respectively. Immune checkpoint conditions were compared with the control conditions without antibody using Dunn’s multiple comparison post hoc test. Two-tailed Student’s *t* test was used to assess the significance for data summarized in Fig. 6. The *p* values below 0.05 were considered significant (*), *p* < 0.01 (**), *p* < 0.001 (***), and *p* < 0.0001 (****).

## Results

### CD4^+^ T-cell responses to TT

**Fig. 1:**
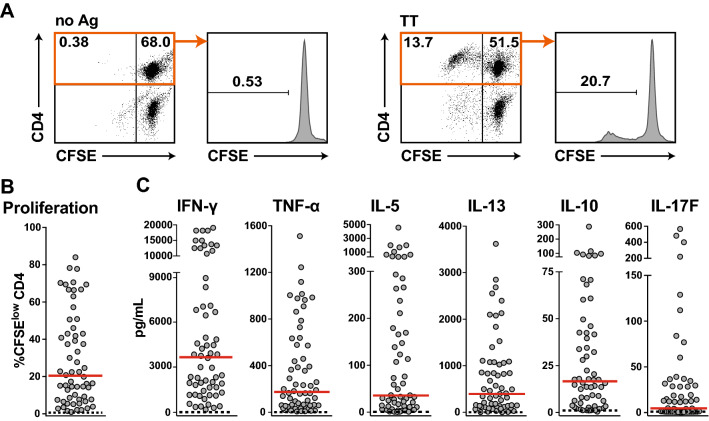
CD4^+^ T-cell responses to tetanus toxoid (TT). **a** Dot plots depict CFSE versus CD4 of live cells and histograms show percentage of live CD4^+^ CFSE^low^ T cells of a representative experiment. **b** Percentage of CFSE^low^ CD4^+^ T cells of 63 study donors are shown. **c** The concentration of the indicated cytokines of each stimulated donor sample is represented by a single dot. **b**+**c** Dashed lines indicate values for unstimulated conditions. Median values are shown in red

PBMCs were isolated from 65 donors, labeled with CFSE, and stimulated with 10 Lf/mL TT for 6–7 days (Fig. 1a). Sixty-three of these donors specifically responded to this antigen and strong proliferation of CD4^+^ T cells was observed in a majority of the samples. The percentage of CFSE^low^ CD4^+^ T cells ranged from 1 to 84% (median 20.4%) (Fig. 1a, b). Cytokine responses of the PBMC cultures were measured using LEGENDplex™-based multiplexing. Supernatants of TT-stimulated cultures contained high concentrations of the T_H_1 cytokine IFN-γ (median concentration of 3.8 ng/mL) as well as the T_H_2 cytokine IL-13 (median concentration 450 pg/mL), whereas the levels of TNF-α, IL-17F, and IL-10 were low (Fig. 1c). The median proliferation and cytokine production were very low in PBMCs cultured in the absence of TT (Fig. 1).

### Expression of PD-1, LAG-3, BTLA, and CTLA-4 on T cells

**Fig. 2:**
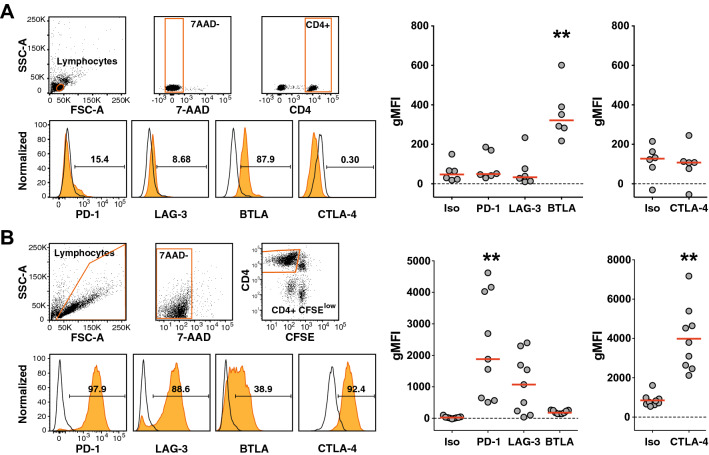
Regulation of PD-1, LAG-3, BTLA and CTLA-4 on CD4^+^ T cells. **a** Unstimulated CD4^+^ T cells of healthy donors were analyzed for the expression of the indicated inhibitory receptors. Gating strategy for viable (7-AAD negative) CD4^+^ T lymphocytes is depicted (upper left panels). Histograms show the expression of immune checkpoints of a representative donor and numbers indicate percent receptor-positive cells (lower left panels). Cumulative data of geometric mean fluorescence intensity (gMFI) of six donors are shown in the scatter dot plot (right). **b** CFSE-labeled PBMCs of nine donors were stimulated with TT. 7-AAD-negative CFSE^low^ CD4^+^ T lymphocytes were analyzed for the expression of the indicated receptors as shown in the histograms for one representative donor and in cumulative scatter plots. **a**, **b** Open histograms represent staining with isotype control antibodies, histograms shown in orange represent antibody staining of the indicated molecules

To assess the regulation of immune checkpoints on human CD4^+^ T cells responding to antigen, we studied the expression of the immune checkpoints PD-1, LAG-3, BTLA, and CTLA-4 in freshly isolated T cells, along with T cells that had proliferated in response to TT. Freshly isolated CD4^+^ T cells contained a large subset of BTLA^+^ cells and a small subset of PD-1^+^ cells. However, expression of LAG-3 and CTLA-4 was not detected (Fig. 2a). TT stimulation induced strong upregulation of PD-1, LAG-3, and CTLA-4, whereas the expression of BTLA was slightly downregulated (Fig. 2b).

### Effect of immune checkpoint blockade on CD4^+^ T-cell responses to TT in vitro

**Fig. 3:**
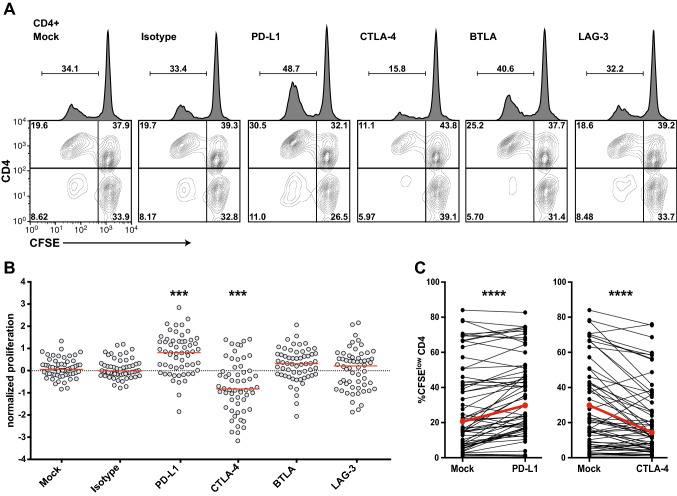
Effect of immune checkpoint blockade on CD4^+^ T-cell proliferation in response to TT. CFSE-labeled PBMCs were stimulated with TT in the presence of blocking antibodies to PD-L1, CTLA-4, BTLA or LAG-3. **a** Representative data showing CD4^+^ T-cell proliferation in the presence of immune checkpoint inhibitors as indicated. Percentages display proliferated (CFSE^low^) CD4^+^ T cells among all live cells (histograms) or proportion thereof in the quadrants (contour plots). **b** Effect of immune checkpoint inhibitors on normalized proliferation scores. **c** Comparison of percentages of CFSE^low^ CD4^+^ T cells in the absence or presence of antibodies to PD-L1 or CTLA-4

In the next step, we evaluated the capability of immune checkpoint inhibitors to enhance the human CD4^+^ T-cell responses to TT in vitro. CFSE-labeled PBMCs stimulated in the presence of blocking antibodies targeting PD-L1, CTLA-4, BTLA, and LAG-3 for 6–7 days were analyzed by flow cytometry. Data of a representative donor are shown in Fig. 3a. Addition of a PD-L1 blocker strongly augmented T-cell proliferation in response to TT (Fig. 3b, c). In pilot experiments, we have tested antibodies to PD-1 and PD-L1. Both reagents strongly enhanced CD4^+^ T-cell proliferation in response to TT (Fig. S1). Since a trend of higher T-cell proliferation was recorded upon addition of the PD-L1 antibody, we have chosen this reagent to block PD-1 signaling. We observed slightly enhanced proliferative effects in the presence of antibodies to BTLA or LAG-3, but this effect did not reach statistical significance (Fig. 3b). Co-blockade of PD-L1 and BTLA or LAG-3 did not promote enhanced proliferation compared to that by blockade of PD-L1 alone (Fig. S2). Surprisingly, the therapeutic CTLA-4 antibody ipilimumab strongly reduced the percentage of CFSE^low^ CD4^+^ T cells in the TT-stimulated cultures (Fig. 3b, c).

**Fig. 4:**
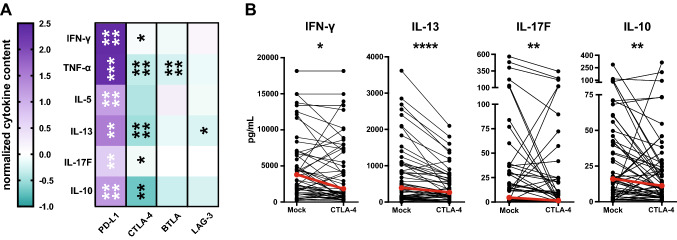
Effect of immune checkpoint blockade on the cytokine production of TT-stimulated PBMC cultures. **a** Normalized cytokine scores of CD4^+^ T cells stimulated in the presence of antibodies against the indicated molecules are shown as a heat map. The median values of IFN-γ, TNF-α, IL-5, IL-13, IL-17F, and IL-10 for the cohort are depicted. **b** Concentration of cytokines in cultures stimulated in the absence and presence of the CTLA-4 antibody ipilimumab

The effect of immune checkpoint blockade on cytokine production was evaluated by multiplex analysis of 13 T-helper cell cytokines in the culture supernatants. In general, IL-2 and IL-4 concentrations were below the limit of detection. Also, levels of IL-9, IL-17A, IL-21, and IL-22 were quite low and could not be significantly enhanced by immune checkpoint blockade (data not shown). Upon blockade of PD-1 signaling, the production of IFN-γ, TNF-α, IL-5, IL-13, IL-17F, and IL-10 was potently enhanced (Fig. 4a). Blockade of BTLA or LAG-3 did not increase the cytokine production in response to TT. The addition of BTLA antibodies strongly reduced TNF-α levels. This phenomenon could probably be explained by the fact that in our stimulation cultures, this cytokine is mainly produced by monocytes/macrophages. These cells are HVEM positive (data not shown) and interference with HVEM engagement by BTLA might reduce TNF-α production by these cells.

Presence of the CTLA-4 antibody ipilimumab also reduced the production of T_H_1 (IFN-γ, TNF-α), T_H_2 (IL-13), and T_H_17 (IL-17F) cytokines along with IL-10 in response to TT. The decline was most pronounced for TNF-α (*p* < 0.0001) and IL-13 (*p* < 0.0001) (Fig. 4).

### Ipilimumab-IgG1, but not ipilimumab-IgG4, impairs effector CD4^+^ T-cell responses in vitro

**Fig. 5:**
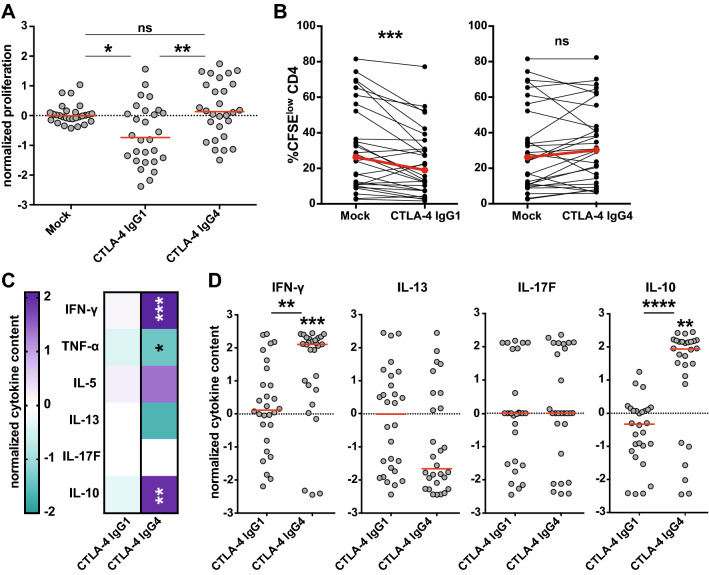
Effect of CTLA-4 antibody isotype on CD4^+^ T-cell responses. CFSE-labeled PBMCs (*n* = 28) were stimulated with TT in the absence (mock) or in the presence of CTLA-4 antibody ipilimumab variants of different isotypes (IgG1 or IgG4). **a** Normalized proliferation scores of stimulated PBMC cultures. **b** Comparison of mean CFSE^low^ CD4^+^ T-cell percentages upon stimulation in the presence of the indicated CTLA-4 antibodies. **c** Heat map representing median normalized cytokine content of IFN-γ, TNF-α, IL-5, IL-13, IL-17F, and IL-10. **d** Scatter plots showing cytokine concentrations in the TT-stimulated cultures in the presence of different CTLA-4 antibodies

The mechanisms of action of ipilimumab are not fully comprehended. Recent reports indicate that CTLA-4 antibodies can mediate cytotoxic effects on T cells, particularly Tregs, which express high levels of CTLA-4 [16, 21]. Several lines of evidence suggest that ipilimumab, which is an IgG1 antibody, exerts cytotoxic effects on CTLA-4-expressing cells via engagement of Fc receptors on NK cells, monocytes and macrophages [15, 18]. Consequently, we investigated whether the Fc part of ipilimumab mediates the observed reduction in CD4^+^ T-cell responses. For this purpose, we compared the effect of ipilimumab-IgG1 and an IgG4 variant in TT-stimulated PBMC cultures. IgG4 antibodies are characterized by a weak interaction with Fc receptors and a lack of effector functions [22]. Indeed, we observed that using the conventional ipilimumab, which is an IgG1 isotype, resulted in a reduction in proliferated CD4^+^ T cells, whereas the presence of an IgG4 isotype variant induced a slight increase in CD4^+^ T-cell proliferation (Fig. 5a, b). Moreover, we observed that ipilimumab IgG4, but not ipilimumab IgG1, induced significantly enhanced cytokine levels in response to TT (Fig. 5c, d).

### Depletion of CD16^+^ cells abrogates the inhibitory effect of ipilimumab

**Fig. 6:**
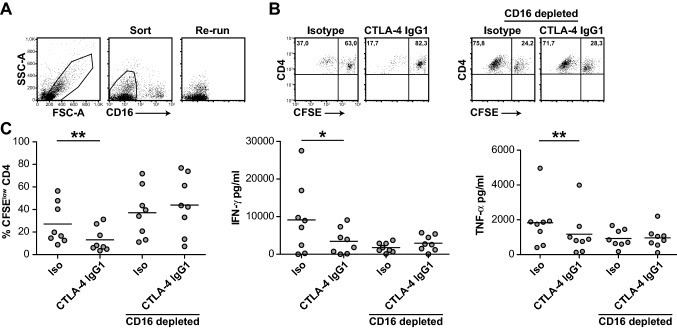
The inhibitory effect of ipilimumab depends on CD16^+^ cells. PBMCs derived from healthy donors (*n* = 8) were stained with a CD16 mAb and depleted of CD16-expressing cells by flow cytometry. **a** Gating strategy and purity of sorted cells from one representative experiment. **b** Dot plots of TT-stimulated cultures of unsorted and CD16^+^ cell-depleted PBMCs of a representative donor. **c** Unsorted and CD16^+^ cell-depleted PBMCs were CFSE-labeled and stimulated with TT in the presence of ipilimumab (CTLA-4 IgG1) or isotype control. Subsequently, CFSE^low^ CD4^+^ T cells and cytokine contents were determined

The results of a previous study have demonstrated that ipilimumab can engage FcγRIIIA (CD16)-expressing non-classical monocytes ex vivo and cause antibody-dependent cellular cytotoxicity (ADCC) of regulatory T cells [18]. To examine whether CD16-expressing cells participate in the impairment of CD4^+^ T-cell responses by ipilimumab, we used flow sorting to deplete CD16^+^ cells from PBMCs (Fig. 6a). Comparison of CD16^+^ cell-depleted and unsorted PBMCs demonstrated that the inhibitory effects of ipilimumab on CD4^+^ T-cell proliferation and cytokine production were dependent on the presence CD16-expressing cells (Fig. 6b, c). Taken together, our results indicate that the therapeutic CTLA-4 antibody ipilimumab can impair CD4^+^ effector T-cell responses. Moreover, this activity relies on its IgG1-Fc part and CD16-expressing cells.

## Discussion

In our study, we have compared blocking antibodies targeting four major inhibitory immune checkpoints (PD-1, BTLA, LAG-3, and CTLA-4) in terms of their capability to enhance CD4^+^ T-cell responses to TT in vitro. Although we found that each of these receptors is expressed on CD4^+^ T cells, which responded to antigen stimulation, a significant enhancement of proliferation and cytokine production was only seen during disruption of PD-1 inhibition using antibodies against PD-1 or PD-L1 (Figs. 3, 4 and Fig. S1). This is in line with numerous previous studies, which report a sturdy increase in the T-cell responses upon PD-1 blockade. Besides, this result highlights the unique potential of PD-1 as a therapeutic target to enhance T-cell responses against pathogen- or tumor-derived antigens [10, 11, 23–26]. Anti-LAG-3 or anti-BTLA antibodies had limited effects on the T-cell responses in our study. The main potential of antibodies targeting emerging immune checkpoints like LAG-3 or BTLA may be realized upon coupling their use with PD-1 blockers. Several studies including work by our group have demonstrated that blocking multiple immune checkpoints can be more effective than blocking PD-1 alone [9–11, 27, 28]. However, we did not observe an enhanced response of TT-specific CD4^+^ T cells upon combined blocking of immune checkpoints compared to blocking PD-1 alone (Fig. S2).

We found that the presence of the therapeutic CTLA-4 antibody ipilimumab substantially decreased the number of proliferated CD4^+^ T cells and lowered the cytokine production in response to TT. This finding was coherent with an earlier study performed by us that demonstrated an impaired in vitro proliferation and cytokine production of allergen-specific CD4^+^ T cells in the presence of ipilimumab [20]. Although CTLA-4 was the first immune checkpoint successfully targeted clinically and its inhibitory role in T-cell responses is clearly established, the mechanisms of CTLA-4-mediated T-cell suppression are still not well understood [29]. Initially, the inhibitory signaling mediated via the cytoplasmic tail of CTLA-4 was examined [30–34], but there is also evidence that this receptor limits T-cell responses in an extrinsic manner [35, 36]. It can prevent costimulatory signaling by outcompeting the binding of CD28 to the B7 molecules and by depleting B7 molecules from APCs through the process of transendocytosis [37, 38]. We have previously used CTLA-4 variants lacking a cytoplasmic domain in a T-cell reporter system and found that they are fully competent to inhibit T-cell responses, which corroborates the vital role of extrinsic effects on CTLA-4 function [39]. CTLA-4 is strongly expressed in Treg cells and it possibly inhibits T-cell responses via them. Importantly, several recent reports have otherwise suggested that, in vivo, CTLA-4 antibodies function by depleting intra-tumoral Tregs rather than by blocking CTLA-4 [16, 21]. Ipilimumab has been shown to engage FcγRIIIA (CD16)-expressing non-classical monocytes resulting in ADCC-mediated lysis of Tregs [18]. Based on this observation, the response to ipilimumab treatment was seen to be correlated with the levels of CD16^+^ monocytes and the CD16a-V158F high-affinity polymorphism, implying that depletion of Tregs by CD16^+^ cells might indeed contribute to the efficacy of ipilimumab treatment [15, 18].

There is a surprising lack of in vitro studies reporting robust stimulatory effects of ipilimumab on primary human T cells, whereas numerous reports have documented that PD-1 blockade strongly enhances human CD4^+^ and CD8^+^ T-cell responses in vitro. Previous studies of our group show a tendency of reduced CD4^+^, but not CD8^+^, T-cell responses to allogeneic DC or HIV peptides using ipilimumab, although this effect was not statistically significant in the tested cohorts [10, 11]. Moreover, we have found that ipilimumab induced a profound reduction in CD4^+^ T-cell proliferation and cytokine production in PBMC cultures stimulated with allergen-containing extracts [20]. In the current study, we demonstrate that ipilimumab significantly reduced CD4^+^ T-cell responses to TT in a large cohort of donors. Since this effect was specifically observed with ipilimumab–IgG1, but not with an IgG4 variant of this antibody, we hypothesized that cytotoxic effects might have resulted in depletion of CTLA-4^high^ CD4^+^ responder T cells in our PBMC cultures. To support our hypothesis, we used PBMC cultures depleted of CD16^+^ cells and demonstrated that the inhibitory effect of ipilimumab was abrogated in such cultures. Increasing evidence suggests that Tregs, which express high levels of CTLA-4, are subject to ADCC mediated by CTLA-4 antibodies including ipilimumab [15, 16, 18, 21]. To our knowledge, this is the first study to present data indicating that ipilimumab might also target CD4^+^ effector T cells. Romano et al. co-cultured flow sorted CD4^+^ T cells with CD16^+^ monocytes. They showed that CD25^high^ CD4^+^ T cells had the highest expression of CTLA-4 as well as FOXP3 and revealed that these cells, which were most likely Tregs, were killed in the presence of ipilimumab [18]. In line, our experiments illustrate that upon strong upregulation of CTLA-4, stimulated CD4^+^ effector T cells can also become targets of ipilimumab-mediated ADCC. We assume that ipilimumab will also deplete Tregs in our assay, but their low numbers in PBMC fractions will not significantly affect the CD4^+^ T-cell response and the net effect of this antibody might be inhibitory in vitro. Further efforts are needed to address whether ipilimumab can deplete CTLA-4-expressing CD4^+^ effector T cells in vivo. CTLA-4 expression is lower on CD8^+^ than on CD4^+^ T cells and our previous studies did not indicate impairment of CD8^+^ T-cell response by ipilimumab [10, 11, 40]. This outcome leads us to believe that the depletion of activated CD8^+^ effector T cells in vivo by this antibody seems less likely.

## Conclusion

The results of our study emphasize that during the in vitro evaluation of antibodies targeting inhibitory receptors, it is important to distinguish between direct immune checkpoint inhibition and indirect effects on T-cell responses that are mediated via the interaction with Fc receptors expressed on ADCC-competent cells. Currently, there is great awareness that the isotype of immunomodulatory antibodies critically affects their efficacy in animal studies as well as in the clinic [41–43]. Our study indicates that the IgG1 antibody ipilimumab also mediates killing of activated effector CD4^+^ T cells in vitro and thus extends previous findings that this therapeutic antibody can exert cytotoxic effects on Tregs. Taken together, these results strongly suggest that CTLA-4 antibodies, which efficiently block CTLA-4, but do not induce ADCC, might have a very distinct functional profile. Thus, testing the therapeutic potential of such antibodies is warranted.

### Electronic supplementary material

Below is the link to the electronic supplementary material.

Supplementary material 1 (PDF 382 kb)

## Abbreviations

7-AAD: 7-Aminoactinomycin D
ADCC: Antibody-dependent cellular toxicity
BTLA: B- and T-lymphocyte attenuator
CFSE: Carboxyfluorescein succinimidyl ester
HVEM: Herpes virus entry mediator
LAG-3: Lymphocyte activation gene 3
TT: Tetanus toxoid
