# Supplementary material for: CTLA-4 antibody ipilimumab negatively affects CD4+ T-cell responses in vitro
Source: Cancer Immunol Immunother. 2019 Jul 22;68(8):1359–68. doi: 10.1007/s00262-019-02369-x (PMC6683241; doi:10.1007/s00262-019-02369-x)
Supplement: Supplementary file 1 — Supplementary material 1 (PDF 382 kb) [file 262_2019_2369_MOESM1_ESM.pdf]

Supplementary Figures

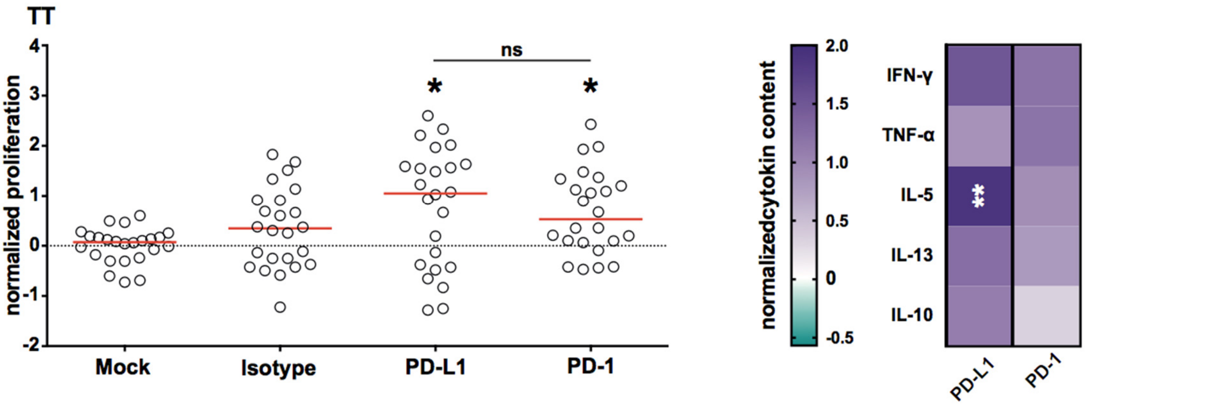

Supplementary Figure 1: *Comparison of blocking antibodies to PD-L1 and PD-1 on CD4⁺ T cell proliferation*

Normalized proliferation scores (left) and median of normalized cytokine contents (right) of CFSE-labeled PBMC cultures stimulated with tetanus toxoid (TT) for 6-7 days in absence or presence of indicated blocking antibodies are shown. Each data point represents the mean of triplicates of one donor and median normalized proliferation for each group is indicated as red line (n=24). Statistical analysis was performed using Friedman test in comparison to mock control and amongst the groups (*P ≤ 0.05, **P ≤ 0.01).

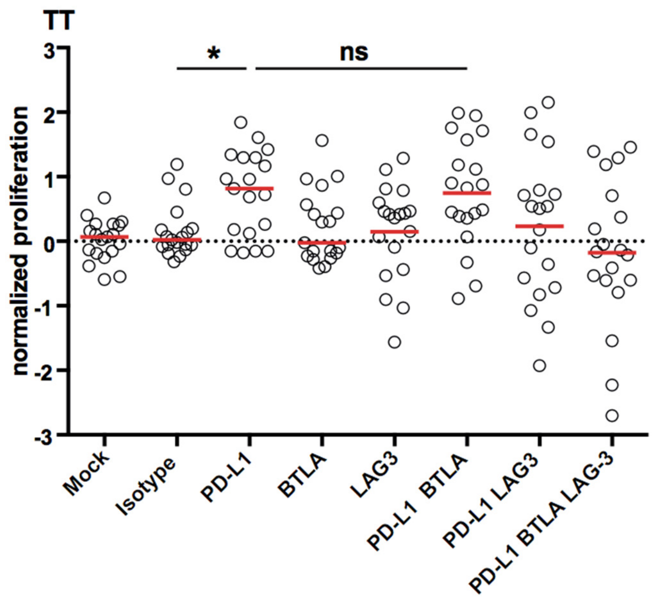

Supplementary Figure 2: *Effect of immune checkpoint inhibitor combinations on CD4⁺ T cell proliferation*

The combinatorial effect of immune checkpoints on T cell proliferation upon stimulation with tetanus toxoid was assessed (n=19). Statistical analysis was performed using Friedman test in comparison to PD-L1 (*P ≤ 0.05).
